# Research on fault diagnosis method of planetary gearbox based on dynamic simulation and deep transfer learning

**DOI:** 10.1038/s41598-022-21339-5

**Published:** 2022-10-11

**Authors:** Meng-Meng Song, Zi-Cheng Xiong, Jian-Hua Zhong, Shun-Gen Xiao, Yao-Hong Tang

**Affiliations:** 1 College of Information, Mechanical and Electrical Engineering, Ningde Normal University, Ningde, China; 2 College of Mechanical Engineering and Automation, Fuzhou University, Fuzhou, China

**Keywords:** Electrical and electronic engineering, Mechanical engineering

## Abstract

To address the issue of not having enough labeled fault data for planetary gearboxes in actual production, this research develops a simulation data-driven deep transfer learning fault diagnosis method that applies fault diagnosis knowledge from a dynamic simulation model to an actual planetary gearbox. Massive amounts of different fault simulation data are collected by creating a dynamic simulation model of a planetary gearbox. A fresh deep transfer learning network model is built by fusing one-dimensional convolutional neural networks, attention mechanisms, and domain adaptation methods. The network model is used to learn domain invariant features from simulated data, thereby enabling fault diagnosis on real data. The fault diagnosis experiment is verified by using the Drivetrain Diagnostics Simulator test bench. The validity of the proposed means is evaluated by comparing the diagnostic accuracy of various means on various diagnostic tasks.

## Introduction

Planetary gearbox (PG) is a critical part of rotating machines, in virtue of its merit of great carrying capacity, small volume, and high driving efficiency, it has been more and more extensively applied in mechanical transmission systems of wind power, aviation, lifting and transportation, and other industries^1^. However, the working environment of PG is usually very harsh, such as often changes in the working environment, heavy loads, bad weather, and many other factors, making it easy to fail. Once the PG fails, it may cause mechanical equipment downtime. Health monitoring and fault diagnosis of PG can keep the mechanical equipment running safely and stably, and reduce possible losses caused by faults.

The conventional fault diagnosis means comprises three steps: data collection, feature extraction, and fault classification^2^. First, the data during the operation of the mechanical equipment is collected by sensors, and then the features are manually picked up from the collected data. Finally, the extracted features are used to train machine learning models such as SVM^3^, KNN^4^, RF^5^, and ANN^6^ for failure prediction. However, manual feature extraction has obvious disadvantages. It requires a lot of manpower and some professional basic knowledge.

Deep learning (DL) is a major breakthrough in artificial intelligence in recent decades. It can automatically pick out representative features from raw data, and accurately build nonlinear mapping relationships of different health status features, which greatly surmounts the deficiencies of traditional fault diagnosis means. DL models such as CNN^7^, AE^8^, DBN^9^, and RNN^10^ have been diffusely researched and utilized in the fault diagnosis field.

When making use of DL means for fault diagnosis, the following two assumptions usually need to be satisfied: (1) sufficient labeled data is available; (2) the feature distribution in the training data and the test data are identical. However, in actual production, majority of data are gathered when the machines is running healthy^11^, so it is very hard to obtain comprehensive and extensive labeled failure data.

In view of the scarcity of fault data, dynamic simulation analysis may be a good solution. The dynamic simulation analysis and diagnostic means frequently begins with the creation of a dynamic model of machines to simulate dynamic response of machines under multiple conditions. Then, the dynamic response may be used to do diagnostics^12^. Xiao et al.^13–15^ revealed the dynamic response of mechanical system under the influence of rub impact fault by establishing a dynamic model. Han et al.^16^ built a revised lumped parameter dynamic model, which revealed the fluctuation of the dynamic load distribution coefficient when gear crack faults were considered. Chen et al.^17^ developed an analytical model for meshing stiffness calculation using the potential energy principle and uniform bending Timoshenko beam theory, and demonstrated the effect law of flexible ring gear and tooth root cracking fault on dynamic response. Park et al.^18^ designed a lumped parameter model of planetary gear dynamics to extract transmission error signal, and applied the rotation error signal to fault diagnosis. Fan et al.^19^ built a dynamic model of the planetary gear train with planetary carrier crack fault, and demonstrated a correlation between vibration characteristics and planetary carrier crack conditions. Duan et al.^20^ used a system-level rigid-flexible coupled model consisting of shells, gears, shafts and bearings to detect the crack evolution-induced vibration, and acquired relevant spectral characteristics, statistical indicators and instantaneous energy of the crack evolution-induced vibration.

By constructing a dynamic model of the mechanical equipment to be diagnosed and solving it numerically, the simulation data of various working conditions and common fault types can be obtained easily, which solves the problem of insufficient real faults. However, limited by the complexity of the simulation model and calculation, the simulation data obtained are often too ideal, lacking noise in the actual environment, which is significantly different from the real data. Therefore, the fault diagnosis model built by the simulation data set will not attain a better precision on the real data set. Transfer learning (TL) is a useful way to settle the matter of data discrepancy.

The purpose of TL is to adapt acquired from one or more tasks (source domain) to other associated but distinct tasks (target domain). It is inspired by the human ability to reuse knowledge from previous tasks to new ones without having to start from the beginning. He et al.^21^ proposed an enhanced depth transmission automatic encoder to address the issue of inadequate bearing vibration data with marks, which initialized the target model through the source model's trained parameter. Then only just a small subset of target training samples are used to fine-tune the target model to fit the features of the remaining target test samples. Li et al.^22^ put forward a joint attention feature transfer network model. The established feature transfer module transfers the generalized representation obtained from the class with more samples to the class with few samples, thus expanding its feature space and solving the matter of data imbalance. Zhu et al.^23^ calculated the domain loss between the source domain and the target domain through the linear combination of multiple Gaussian kernels, which enhanced the adaptive ability of the diagnostic model. Wan et al.^24^ put forward a TL means combining sensitive feature selection and sparse automatic encoder, which reduces the interference of insensitive information in the original signal and raises the diagnostic precision of the model under complex operating conditions. Han et al.^25^ extended the marginal distribution adaptation to joint distribution adaptation, allowing the suggested network to employ the identification structure linked to the labeled data in the source domain to adapt to the conditional distribution of the unlabeled target data and ensure higher precise distribution matching. Zheng et al.^26^ put forward a normalized recursive dynamic adaptive network, which can estimate the relative weight of marginal and conditional distributions dynamically and quantitatively. Deng et al.^27^ put forward a sample weighted joint adversarial network that takes use of the classification information to improve the joint domain adaptability of adversarial learning. Han et al.^28^ proposed a new framework to address the issue of transfer diagnosis for sparse target data. Individual domain adaptation is performed on the source domain data and target data under the same work conditions to reduce distribution differences and avoid the negative transfer.

Aiming at the scarcity of marked fault data in practical fault diagnosis, taking PG as the research object, a fault diagnosis means based on dynamic simulation and deep transfer learning (DTL) is put forward in this paper. By building the dynamic simulation model of the PG, sufficient fault simulation data can be gathered. On this basis, the time-domain graphs (TDG) and frequency-domain graphs (FDG) of the simulation data are evaluated to confirm the validity of established models. In addition, the influence law of various simulation step sizes on simulation data is explored. A fresh DTL network model is built. The network model is composed of three parts: one-dimensional convolution neural network, attention mechanism, and domain adaptive method. Through the network model, the fault diagnosis knowledge contained in a simulation data is used in a real PG’s fault diagnosis, and the influence law of simulation data with distinct parameters on the diagnosis precision is explored.

The remaining of this article is arranged as below: “Theoretical background” outlines the relevant theories. “Proposed method” details the suggested means. “Experiment and analysis” studies a practical case. “Conclusions” summarizes this paper.

## Theoretical background

### Dynamics theory based on ADAMS

In ADAMS, the Cartesian coordinates of the rigid body's centroid and the Euler angles representing its orientation are used as generalized coordinates, hence, *q* = [*x*, *y*, *z*, *ψ*, *θ*, *φ*]^*T*^, Eq. (1) is obtained by the energy form of Lagrange equations of the first kind.

1 $$\frac{d}{dt}\left( {\frac{\partial T}{{\partial \dot{q}_{j} }}} \right) - \frac{\partial T}{{\partial q_{j} }} = Q_{j} + \sum\limits_{i = 1}^{n} {\lambda_{i} } \frac{\partial \phi }{{\partial q_{j} }}$$

Here, *T* denotes the kinetic energy indicated in the generalized coordinate of the system, *q*_*j*_ denotes the generalized coordinate, *Q*_*j*_ represents the generalized force in the direction of *q*_*j*_, and the last term of the equation represents the constraint reaction in the direction of *q*_*j*_.

Introducing generalized momentum *p*_*j*_

2 $$p_{j} = \frac{\partial T}{{\partial \dot{q}_{j} }}$$

The dynamic differential–algebraic equations of the system are established by integrating the constraint equations.

3 $$\left. \begin{gathered} \dot{P} - \frac{\partial T}{{\partial q}} + \Phi_{q}^{T} \lambda + H^{T} F \hfill \\ P = \frac{\partial T}{{\partial {\dot{\text{q}}}}} \hfill \\ u = \dot{q} \hfill \\ \Phi \left( {q,t} \right) = 0 \hfill \\ F = f\left( {u,q,t} \right) \hfill \\ \end{gathered} \right\}$$

Here, Φ is the constraint function, Φ_q_ is the Jacobian matrix of the constraint equation, *H* is the coordinate transformation matrix of the external force, *λ* is the Lagrange multiplier, and *F* represents the external force on the system.

There are three different integration schemes in ADAMS to reduce the order of dynamic differential–algebraic equations.

I3 integral format:

4 $$\left. \begin{gathered} \dot{P} - \frac{\partial T}{{\partial q}} + \Phi_{q}^{T} \lambda + H^{T} F = 0 \hfill \\ P = \frac{\partial T}{{\partial \dot{q}}} \hfill \\ u = \dot{q} \hfill \\ \Phi \left( {q,t} \right) = 0 \hfill \\ F = f\left( {u,q,t} \right) \hfill \\ \end{gathered} \right\}$$

SI2 integral format:

5 $$\left. \begin{gathered} \dot{P} - \frac{\partial T}{{\partial q}} + \Phi_{q}^{T} \lambda + H^{T} F = 0 \hfill \\ P = \frac{\partial T}{{\partial \dot{q}}} \hfill \\ u - \dot{q} + \Phi_{q}^{T} \mu = 0;(\mu = 0) \hfill \\ \Phi \left( {q,t} \right) = 0 \hfill \\ \dot{\Phi }\left( {q,u,t} \right) = 0 \hfill \\ F = f\left( {u,q,t} \right) \hfill \\ \end{gathered} \right\}$$

SI1 integral format:

6 $$\left. \begin{gathered} \dot{P} - \frac{\partial T}{{\partial q}} + \Phi_{q}^{T} \dot{\eta } + H^{T} F = 0 \hfill \\ P = \frac{\partial T}{{\partial \dot{q}}} \hfill \\ u - \dot{q} + \Phi_{q}^{T} \dot{\xi } = 0 \hfill \\ \Phi \left( {q,t} \right) = 0 \hfill \\ \dot{\Phi }\left( {q,u,t} \right) = 0 \hfill \\ F = f\left( {u,q,t} \right) \hfill \\ \end{gathered} \right\}$$

### Transfer learning

First, various TL-related symbols are given to properly indicate the issues that need to be resolved. Given the source domain $D_{{s{\text{rc}}}} = \{ (x_{i} ,y_{i} )\}_{i = 1}^{{n_{src} }}$ with *n*_*src*_ labeled samples and the target domain $D_{tar} = \{ (x_{i} )\}_{i = 1}^{{n_{tar} }}$ with *n*_*tar*_ unlabeled samples, where $X = \{ (x_{i} )\}_{i = 1}^{n}$ denotes the feature space and $Y = \{ (y_{i} )\}_{i = 1}^{n}$ denotes the label space. Let *P*(*x*) and *Q*(*y|x*) represent marginal and conditional probability distribution respectively. The goal of TL is to use *D*_*src*_ diagnostic knowledge with *D*_*tar*_ in the case of *X*_*src*_ = *X*_*tar*_, *Y*_*src*_ = *Y*_*tar*_, *P*_*src*_(*x*_*src*_) ≠ *P*_*tar*_(*x*_*tar*_), *Q*_*src*_(*y*_*src*_*|x*_*src*_) ≠ *Q*_*tar*_(*y*_*tar*_*|x*_*tar*_).

### Joint distribution adaptation

The joint distribution adaptation (JDA) method was come up with Long et al.^29^, and its goal is to find a transformation *A* to make the distance between *P*_*src*_*(A*^*T*^*x*_*src*_*)* and *P*_*tar*_*(A*^*T*^*x*_*tar*_*)* as close as possible, and at the same time, make the distance between the transformed conditional probability distribution *Q*_*src*_*(y*_*src*_*|A*^*T*^*x*_*src*_*)*and *Q*_*tar*_*(y*_*tar*_*|A*^*T*^*x*_*tar*_*)* also as close as possible.

The approach is broken down into two stages. First, the maximum mean discrepancy (MMD) would be utilized to judge the disparity between *P*_*src*_*(x*_*src*_*)* and *P*_*tar*_*(x*_*tar*_*)*. The distance between the sample means of *D*_*src*_ sample and *D*_*tar*_ sample can be demonstrated that

7 $$\left\| {\frac{1}{{n_{src} }}\sum\limits_{i = 1}^{{n_{src} }} {A^{T} x_{i} - \frac{1}{{n_{tar} }}\sum\limits_{{j = n_{src} + 1}}^{{n_{src} + n_{tar} }} {A^{T} x_{j} } } } \right\|^{2} = tr\left( {A^{T} XM_{o} X^{T} A} \right)$$

where *tr(·)* stands for a matrix's trace, *X* denotes the input data matrix, *M*_*0*_ represents the MMD matrix and Eq. (8) shows the computation equation.

8 $$(M_{o} )_{ij} = \left\{ {\begin{array}{*{20}c} {\frac{1}{{n_{src} n_{src} }},\quad x_{i} ,x_{j} \in D_{s} } \\ {\frac{1}{{n_{tar} n_{tar} }},\quad x_{i} ,x_{j} \in D_{t} } \\ {\frac{ - 1}{{n_{src} n_{tar} }},\quad otherwise} \\ \end{array} } \right.$$

Then *Q*_*src*_*(y*_*src*_*|x*_*src*_*)* and *Q*_*tar*_*(y*_*tar*_*|x*_*tar*_*)* are adapted, and the distance between the conditional probability distributions can be written as

9 $$\left\| {\frac{1}{{n_{src}^{(c)} }}\sum\limits_{{x_{i} \in D_{src}^{(c)} }} {A^{T} x_{i} - \frac{1}{{n_{tar}^{(c)} }}\sum\limits_{{x_{j} \in D_{tar}^{(c)} }} {A^{T} x_{j} } } } \right\|^{2} = tr(A^{T} XM_{c} X^{T} A)$$

where $D_{src}^{(c)}$ represents the source domain data with category *c*, $D_{tar}^{(c)}$ represents the target domain data with category *c*, *M*_*0*_ represents the MMD matrix involving class labels and its calculation formula is shown in Eq. (10).

10 $$(M_{c} )_{ij} = \left\{ {\begin{array}{*{20}l} {\begin{array}{*{20}l} {\begin{array}{*{20}l} {\frac{1}{{n_{src}^{(c)} n_{src}^{(c)} }},\quad x_{i} ,x_{j} \in D_{src}^{(c)} } \hfill \\ {\frac{1}{{n_{tar}^{(c)} n_{tar}^{(c)} }},\quad x_{i} ,x_{j} \in D_{tar}^{(c)} } \hfill \\ \end{array} } \hfill \\ {\frac{ - 1}{{n_{src}^{(c)} n_{tar}^{(c)} }},\quad \left\{ {\begin{array}{*{20}c} {x_{i} \in D_{src}^{(c)} ,x_{j} \in D_{tar}^{(c)} } \\ {x_{j} \in D_{src}^{(c)} ,x_{i} \in D_{tar}^{(c)} } \\ \end{array} } \right.} \hfill \\ \end{array} } \hfill \\ {{0,}\quad \quad \quad {\text{otherwise}}} \hfill \\ \end{array} } \right.$$

Combining Eqs. (7) and (9), a general optimization objective is obtained

11 $$\min \sum\limits_{c = 0}^{C} {tr(A^{T} } XM_{c} X^{T} A) + \lambda \left\| A \right\|_{F}^{2}$$

When Eq. (11) is the smallest, the JDA distance is the smallest, that is, the disparity between *D*_*src*_ sample and *D*_*tar*_ sample is the smallest.

### Attention mechanism

Attention mechanism is derived from the studies of human vision. When mankind recognizes things, they often focus more on important information and ignore irrelevant information. Different features contribute differently to the final classification results throughout the training phase of a DL model. The attention method allows DL model to concentrate on features that have a significant impacts on classification precision and disregard those that do not matter, improving classification performance. CBAM put forward by woo et al.^30^, is an attention mechanism module that integrates space and channel. The method first assigns various weights to various channels of the input feature through the channel attention module. The weight given by important channels is larger and that given by unimportant channels is smaller. Then, through the spatial attention module, different weights are given to different regions of the input features. The overall calculation process is shown in Eqs. (12) and (13).

12 $$C^{\prime} = M_{{\text{c}}} (C) \otimes C$$

13 $$C^{\prime\prime} = M_{s} (C^{\prime}) \otimes C^{\prime}$$

Among them, *C* represents the input feature map, *C’* represents the feature map processed by the channel attention module, *C’’* represent the output feature map, *M*_*c*_ and *M*_*s*_ denote the channel and spatial attention modules, respectively, and represents the element by element multiplication.

## Proposed method

**Figure 1:**
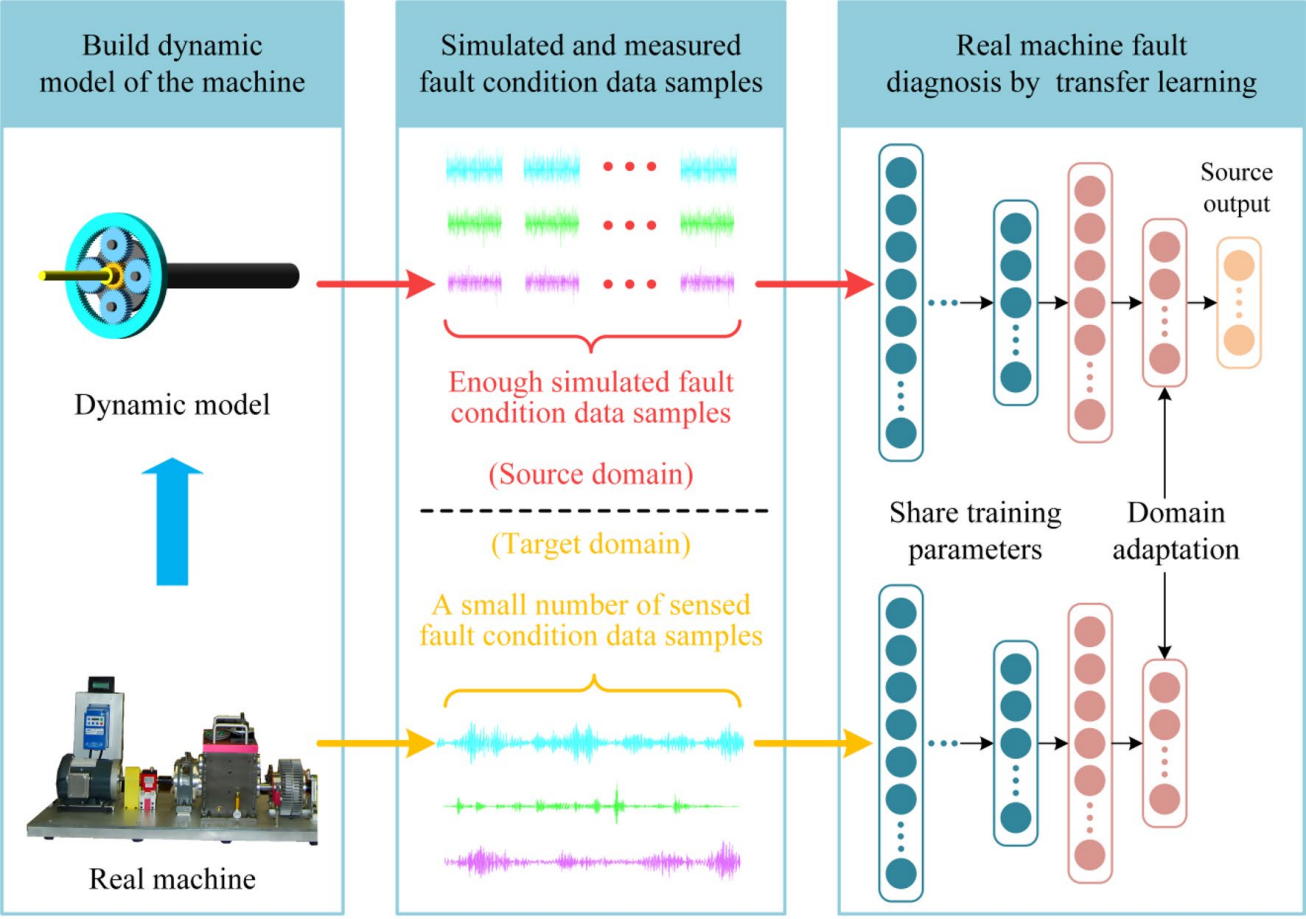
The framework of fault diagnosis means based on dynamic simulation and DTL.

In this paper, a fresh fault diagnosis means for PG is put forward based on dynamic simulation and DTL. This method mainly includes three parts: Building dynamic simulation models of PG, acquiring and analyzing the simulation data and real data, and fault diagnosis of PG based on TL. Figure 1 shows the general framework of the means.

### Building dynamic simulation models of PG

In the light of the sizes of the actual PG related parts, use the 3D modeling software to build a 3D model, and import the 3D model into ADAMS to build a dynamic simulation model.

### Acquiring and analyzing the simulation data and real data

Through the dynamic simulation model, the dynamic response of the actual PG under various fault conditions is simulated, and sufficient simulation data are obtained. By analyzing the TDG and FDG of the fault simulation data, the simulation model's validity is confirmed. By comparing the TDG and FDG of different simulation data, the influence law of simulation step size on simulation data is explored; Collect a small amount of real data from the actual PG.

### Fault diagnosis of PG based on TL

**Figure 2:**
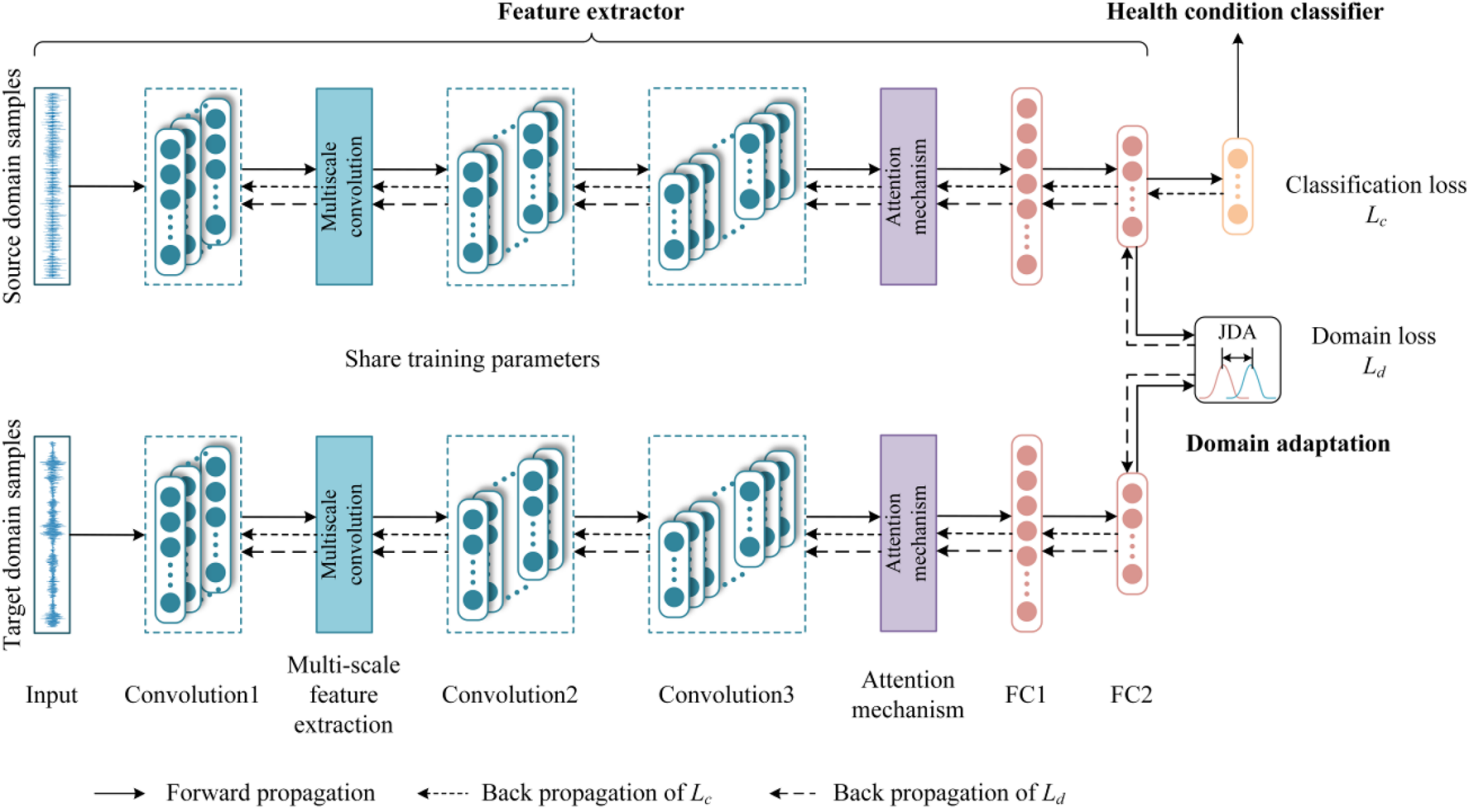
The framework of DTL network model.

**Figure 3:**
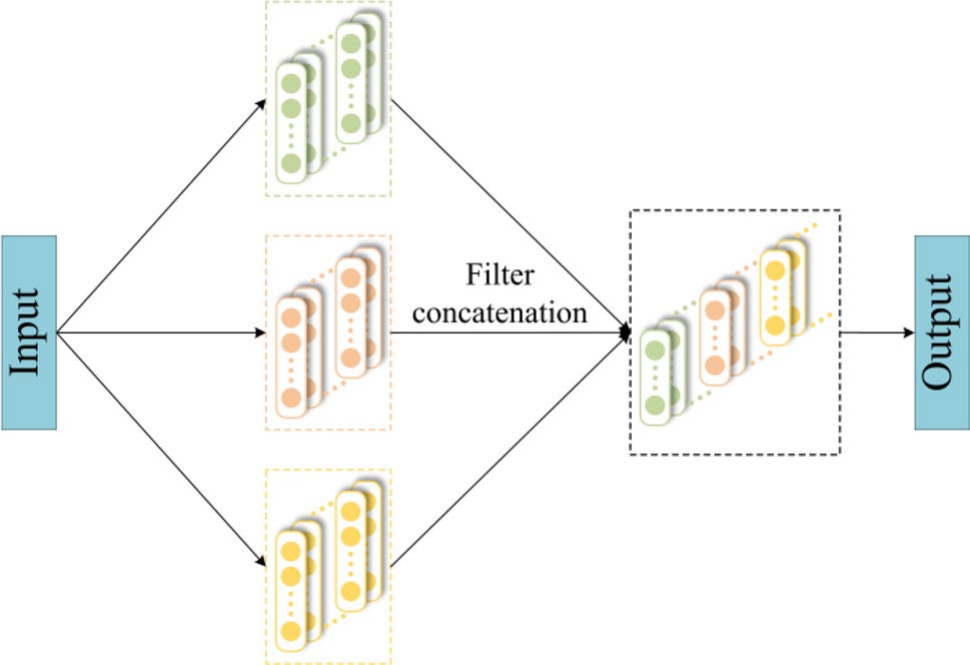
Multi-scale feature extraction layer.

The proposed DTL network model is comprised of three modules: feature extractor, health classifier, and domain adaptation. The model's framework is shown in Fig. 2. The feature extractor is applied to extract high-level abstract features of data. Figure 3 shows the structure of the multi-scale feature extraction layer, which extracts features in parallel using three convolution kernel branches of various scales to obtain features of various scales. By stacking the features of these three scales, a rich multi-scale feature set is formed, and Table 1 displays the feature extractor's network parameters. In the attention mechanism layer, CBAM is applied to enhance important features and suppress irrelevant features; The health status classifier is applied to distinguish health conditions of *D*_*src*_ features. The domain adaptation part adopts joint distribution adaptation (JDA) to execute domain adaptation on *D*_*src*_ features and *D*_*tar*_ features.

**Table 1:** Model network parameters.

Network structure	Number of convolution kernels	Convolution kernel size	Output size
Convolution layer1	16	64	16 × 128
Multiscale feature extraction layer	32	3	16 × 126
		5	
		7	
Convolution layer2	96	3	96 × 124
Convolution layer3	192	3	192 × 122
Full connection layer 1	128	–	128 × 1
Full connection layer 2	3	–	3 × 1

Two optimization objectives must be met during model training:

1. Minimize the classification loss *L*_*c*_ of the feature extractor on *D*_*src*_ data, *L*_*c*_ is calculated by the cross-entropy loss function. Following is the calculating formula:
  14 $$L_{{\text{c}}} = - \sum\limits_{i = 1}^{N} {y_{true}^{i} \log \left( {y_{pred}^{i} } \right)}$$
  where *y*_*true*_ represents the *D*_*src*_ data’s true label, *y*_*pred*_ represents the *D*_*src*_ data’s predicted label.
2. Minimize the domain loss *L*_*d*_, which is calculated by JDA.

By combining the classification loss *L*_*c*_ and the domain loss *L*_*d*_, the overall optimization target of the model can be obtained:

15 $$L = L_{c} + \lambda L_{d}$$

in which, λ Represents the penalty factor. By minimizing *L*, the features learned by the feature extractor can have small domain differences and become domain invariant features, and health condition classifier can accurately classify these features. Take the simulation samples as *D*_*src*_ and the unlabeled real samples as *D*_*tar*_ into the DTL network model, implement fault diagnosis of actual PG, and explore influence laws of simulation data of different parameters on the diagnosis results.

## Experiment and analysis

### Establishment of the dynamic simulation model of PG

**Figure 4:**
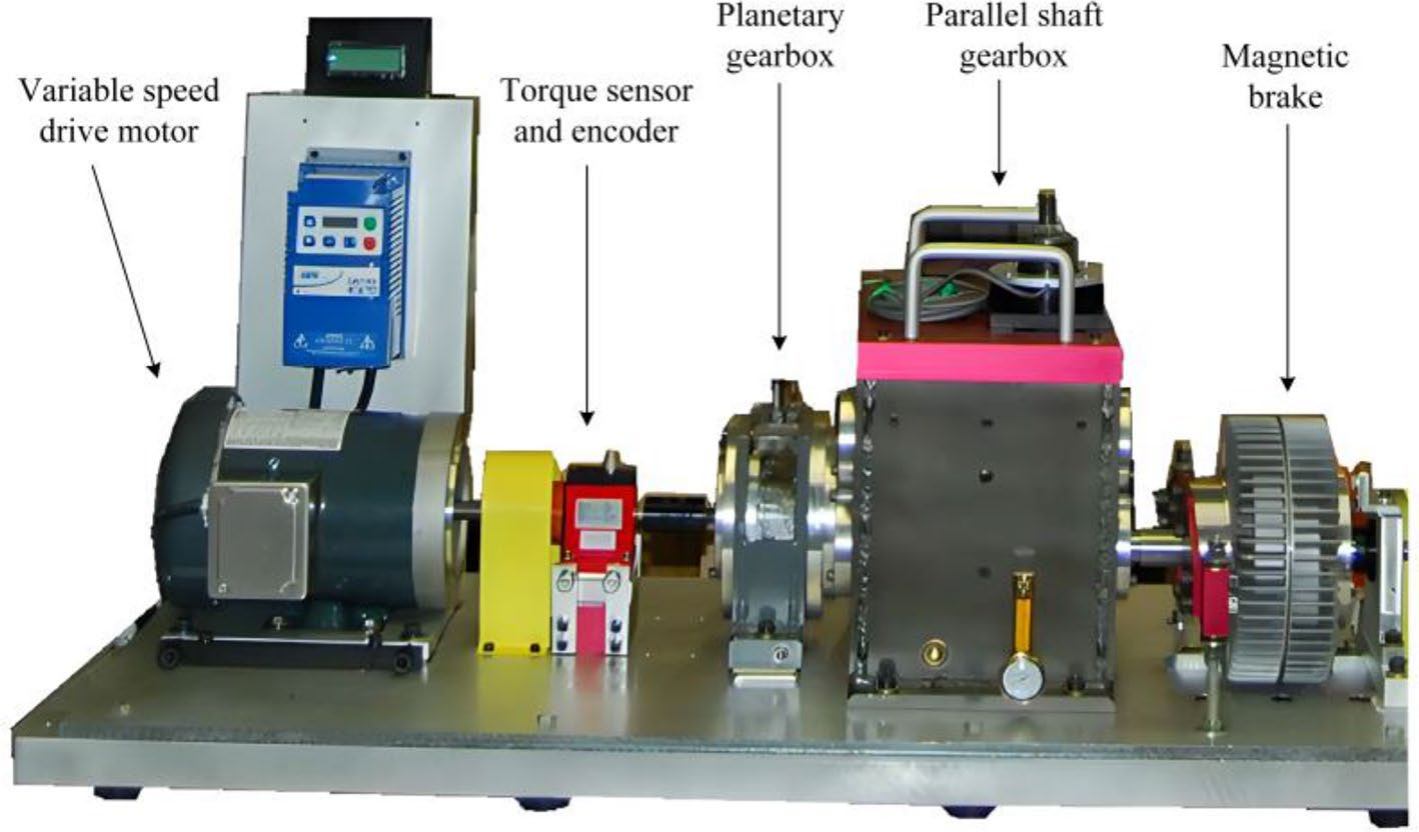
Physical drawing of DDS test bench.

This paper takes the PG in Drivetrain Diagnostics Simulator (DDS) test bench designed by SpectraQuest company of America as the research target, Fig. 4 is the structural drawing of DDS laboratory bench, which is comprised of variable speed drive motor, torque sensor and encoder, PG, parallel shaft gearbox and magnetic brake. Table 2 demonstrates the fundamental parameters of the PG.

**Table 2:** Parameters of the PG.

Part	Quantity of teeth	Modulus	Pressure angle	Tooth width (mm)
Sun gear	28	1	20°	10
Planetary gear	36	1	20°	10
Ring gear	100	1	20°	10

In the light of the parameters demonstrated in Table 2, the three-dimensional model of PG is built and assembled. In order to make the model more clearly reflect the dynamic characteristics, a number of parts that have seldom impact on the simulation results are simplified in the modeling process.

Save the 3D model as x_t format and import it into ADAMS. Considering that the rigid body model ignores the elastic deformation of the parts and cannot accurately transmit the vibration signal, the sun gear is constructed flexible. Import the 3D model of the sun gear into ANSYS, build and export a mode neutral file, replace the rigid body sun gear in ADAMS with this file, and obtain the rigid-flexible coupled model of the PG.

**Figure 5:**
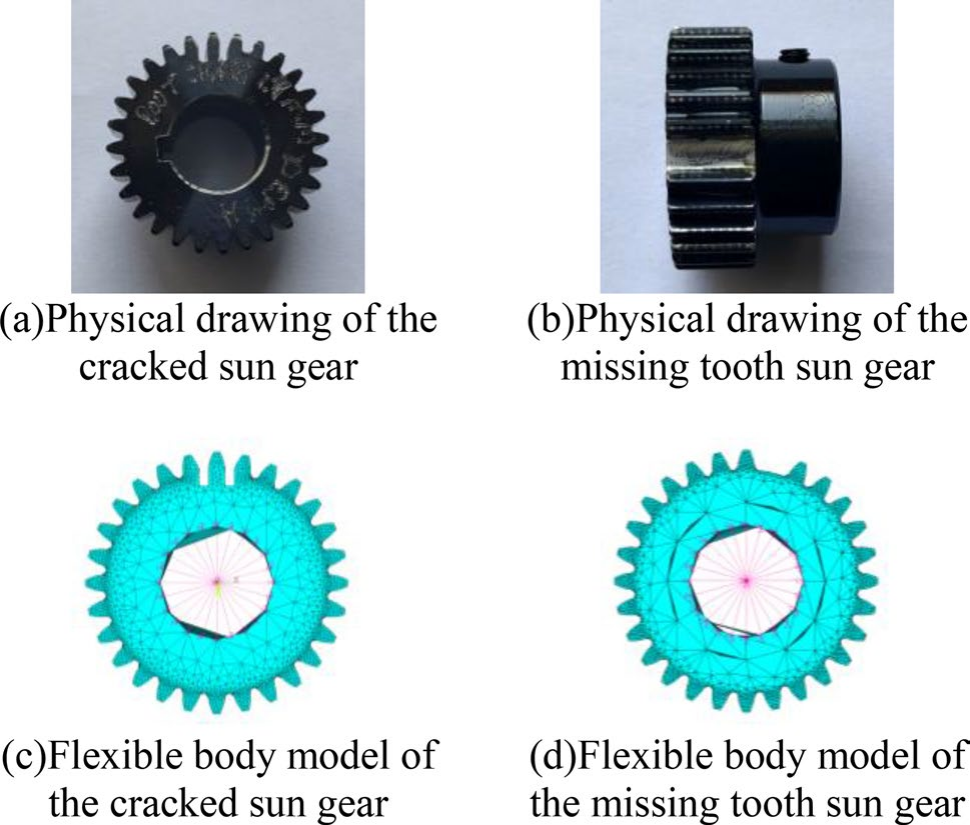
The physical drawing and flexible body model of the cracked and the missing tooth sun gear.

By replacing flexible body sun gears in the rigid-flexible coupled model, the PG simulation models of the sun gear under different health conditions are obtained. Figure 5 shows the physical drawing and flexible body model of the cracked and the missing tooth sun gear.

In line with the actual transmission relationship between the parts, add the constraints shown in Table 3. Since the gear pair can only transmit the speed between the gears and cannot reflect the change of the meshing force between the gears, contact forces are added between the sun gear and the planetary gears and between the planetary gears and the ring gear to replace gear pairs.

**Table 3:** Constraints.

Part 1	Part 2	Constraint
Input shaft	Sun gear	fixed pair
Ring gear	Ground	fixed pair
Input shaft	Ground	Rotary pair
Planetary gear	Planet carrier	Rotary pair
Planet carrier	Ground	Rotary pair

**Figure 6:**
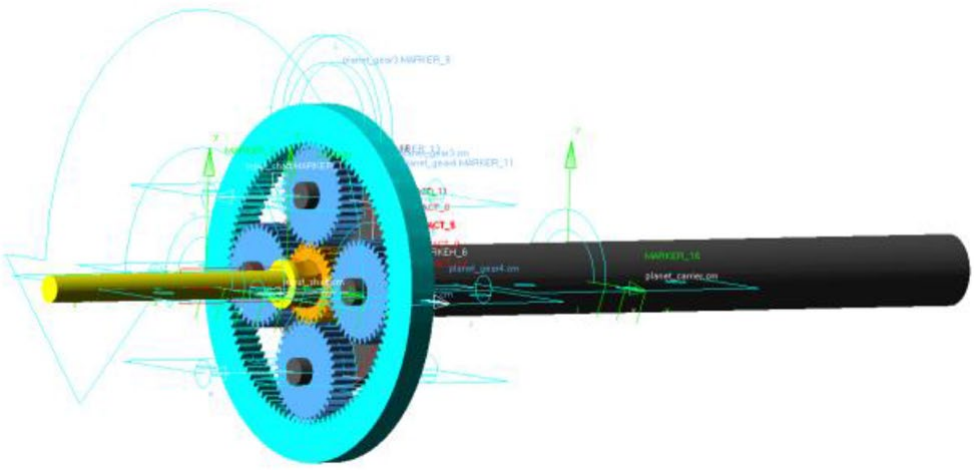
Rigid-flexible coupling model of the PG.

The PG model designed in this paper takes the input shaft as the input, the planetary carrier as the output, and the ring gear is fixed. Therefore, a 30 Hz rotation frequency is added to the input shaft as the speed drive, and the corresponding speed is 10,800°/s. In order to prevent the speed mutation of the input shaft, resulting in infinite acceleration, the step function is used to gradually increase the speed from 0 to 10,800°/s within 0.01 s. Figure 6 demonstrates the completed rigid-flexible coupling model of the PG.

### Acquisition and analysis of simulation data

According to the calculation formula of the transmission ratio of the epicyclic gear train, the calculation formula of the planet carrier rotation frequency *f*_*c*_, planetary gear train meshing frequency *f*_*m*_, and sun gear local fault characteristic frequency *f*_*g*_ can be deduced

16 $$f_{c} = \frac{{Z_{s} \times f_{s} }}{{Z_{s} + Z_{r} }}$$

17 $$f_{m} = f_{c} \times Z_{r}$$

18 $$f_{g} = \frac{{f_{m} \times N}}{{Z_{s} }}$$

Here *Z*_*s*_ stands for the amount of teeth of the sun gear, *Z*_*r*_ stands for the amount of teeth of the ring gear, *f*_*s*_ denotes the rotation frequency of the sun gear, and *N* denotes the amount of planetary gears. Substitute *Z*_*s*_ = 28, *Z*_*r*_ = 100, *f*_*s*_ = 30 Hz, *N* = 4 into Eqs. (14)–(16) to obtain *f*_*c*_ = 6.5625 Hz, *f*_*m*_ = 656.25 Hz, *f*_*g*_ = 93.75 Hz.

When the sun gear's faulty gear teeth mesh with the planetary gear teeth, the lubricating oil film between the sliding contact surfaces of the gear teeth will be ruptured, resulting in an impact phenomenon. The impact will bring about the amplitude modulation and frequency modulation effect on the meshing vibration, and the vibration signal at the meshing point can be described by the amplitude modulation and frequency modulation process. If the integer multiples of meshing frequency *kf*_*m*_ are used as the carrier frequency, the sidebands will appear at *kf*_*m*_ + *mf*_*g*_, and the spacing between the sidebands is *f*_*g*_^31^.

In ADAMS, set the simulation time to 0.5 s, select WSTIFF as the integration solver, and I3 as the integration format. The simulation step size will affect the results of the simulation calculation. If the step length is set too big, the calculation accuracy will be low, and if the step length is set too little, the computing time will be long. Simulate on the basis of the three groups of parameters shown in Table 4 to explore the influence law of simulation step size on simulation data.

**Table 4:** Simulation parameters.

Number	Simulation time (s)	Simulation step size (s)	Simulation data sampling frequency (kHz)
1	0.5	7.8125 × 10^–5^	12.8
2	0.5	1.5625 × 10^–5^	64
3	0.5	8.6806 × 10^–6^	115.2

**Figure 7:**
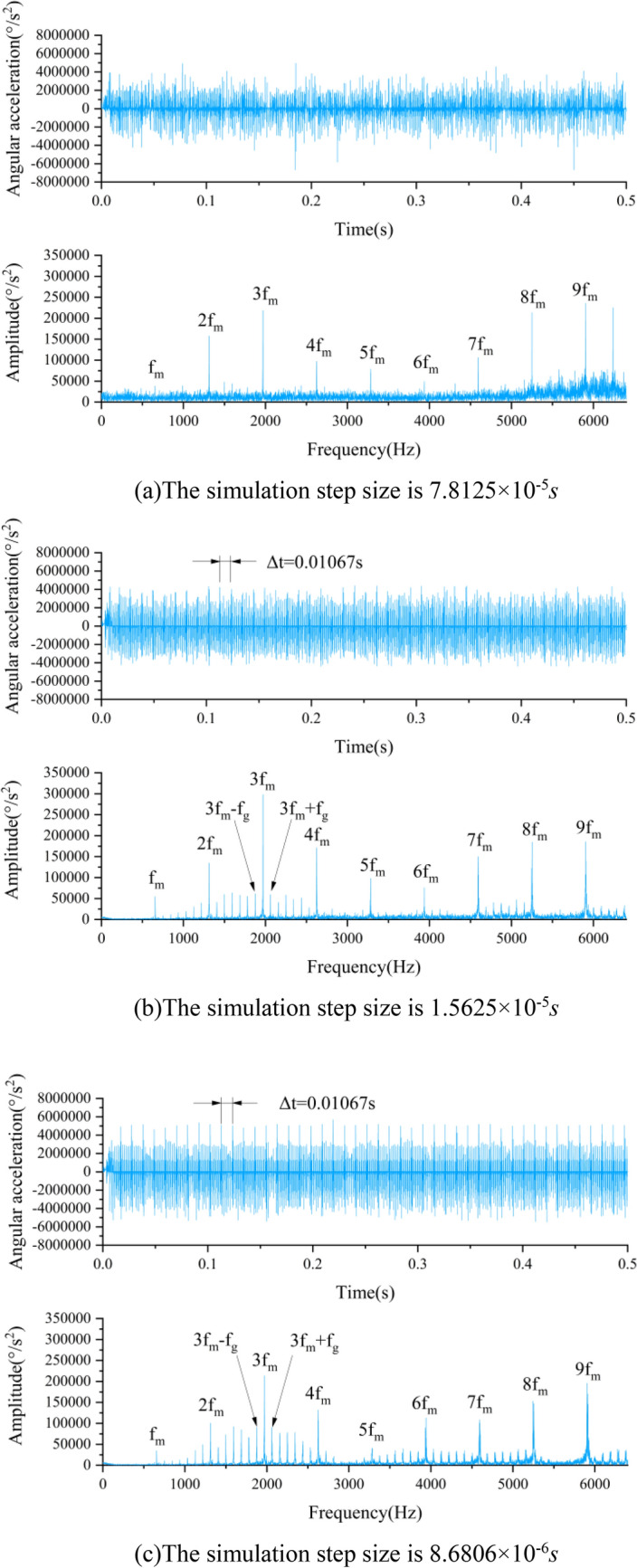
TDG and FDG of different simulation step sizes of rigid-flexible coupling model.

The models used in the simulation are all rigid-flexible coupled models with missing teeth sun gear. After the end of the simulation, angular acceleration on the planet carrier is exported as simulation data. Figure 7 demonstrates TDG and FDG of various simulation data.

As can be aware of the TDG in Fig. 7 that the smaller the simulation step size is, the more obvious the periodic shock phenomenon appears in the TDG, and the time interval between shocks is the same as the theoretical value 1/*f*_*g*_ = 0.01067 s; As can be aware of the FDG of different simulation step size have obvious peaks at *f*_*m*_ = 656.25 Hz and its integer multiples. The smaller the simulation step size is, the more obvious sidebands appear around meshing frequency *f*_*m*_ and its integer multiple in the FDG, and the spacing between the sidebands are all *f*_*g*_ = 93.75 Hz.

**Figure 8:**
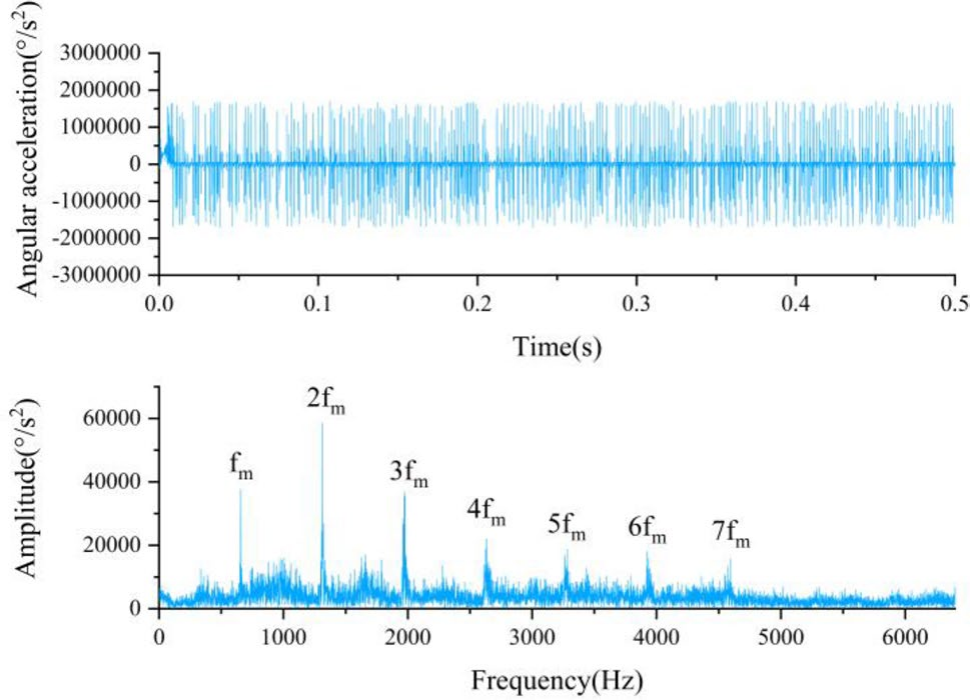
TDG and FDG of the pure rigid body model.

Figure 8 demonstrates the TDG and FDG of the pure rigid body model. The sun gear in the model is missing tooth fault, and the simulation step size is 8.6806 × 10^−6^ s. As can be aware of Fig. 8, the TDG of the rigid body model has no periodic impact, and the FDG has obvious peaks at the meshing frequency and its frequency doubling, but there are no sidebands.

Based on the above comparative analysis, it can be found out that the impact characteristics in the TDG and the fault frequency characteristics in the FDG of the simulation data are consistent with the theory, which certifies the rationality of the dynamic simulation model. Furthermore, contrasted with the pure rigid body model, the rigid-flexible coupling model can better reflect the fault characteristics of the planetary gear train, and the smaller the simulation step size, the clearer the periodic impact in the TDG and the sidebands in the FDG.

### Data description

The real data are the vibration signals acquired from PG of the DDS test bench. When collecting the signal, variable speed drive motor’s rotation frequency is 30 Hz, and there are four different currents on the magnetic brake: 0 A, 0.4 A, 0.8 A, and 1.2 A (different loads can be applied to the output shaft by adjusting the magnetic brake's current). The sun gear contains three health conditions: sun gear cracks, sun gear missing teeth, sun gear normal, and sampling frequency is 12.8 kHz.

The input shaft rotation frequency of the simulation model is 30 Hz and there is no load. After the end of the simulation, angular acceleration on the planet carrier is obtained as simulation data.

The simulation data and real data are overlapping sampling to obtain simulation samples and real samples. 2048 points are sampled every 100 points, that is, 2048 data points per sample. Table 5 displays the number of samples.

**Table 5:** Data set description.

Name	Dataset	Health condition	Number of samples	Load
A	Simulation data	Crack	2000	0
		Missing teeth	2000	0
		Normal	2000	0
B	Real data	Crack	1200	0
		Missing teeth	1200	0
		Normal	1200	0
C	Real data	Crack	1200	0.4
		Missing teeth	1200	0.4
		Normal	1200	0.4
D	Real data	Crack	1200	0.8
		Missing teeth	1200	0.8
		Normal	1200	0.8
E	Real data	Crack	1200	1.2
		Missing teeth	1200	1.2
		Normal	1200	1.2

### Analysis of influence law of simulation parameters

**Figure 9:**
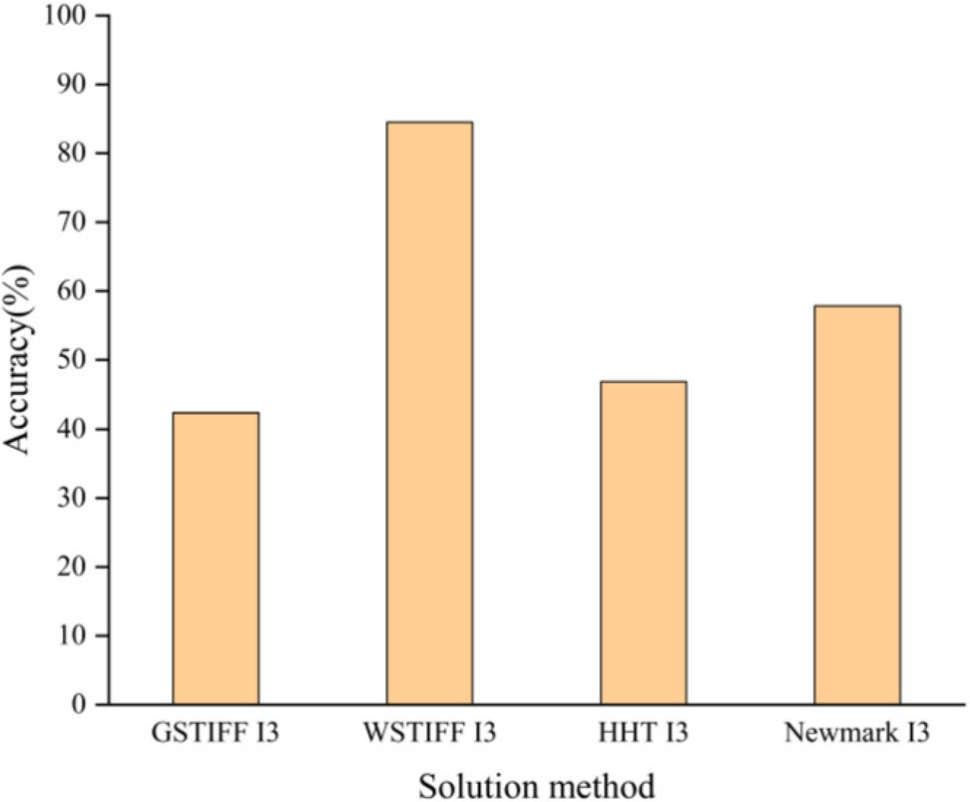
Diagnostic accuracy of simulation data for different solution methods.

There are five different integration solvers in Adams: GSTIFF, WSTIFF, HHT, Newmark, HASTIFF, and three integration formats: I3, SI1, SI2. The characteristics of various solution means are demonstrated in Table 6. Take the simulation data of various solution means as *D*_*src*_ and the real data set B as *D*_*tar*_ for TL. The diagnosis precision is demonstrated in Fig. 9. As can be aware of Fig. 9, when the integration solver selects WSTIFF and the integration format selects I3, the diagnosis accuracy is the highest.

**Table 6:** Characteristics of the different solution means in ADAMS.

Solver	Integral format	Characteristics of solution method	Calculation accuracy and simulation speed
GSTIFF	I3	Estimate-correction error is judged by displacement error	Highest calculation accuracy and fast simulation speed
WSTIFF	I3		
GSTIFF	SI2	Estimate-correction error is judged by speed error	High calculation accuracy, slowest simulation speed
WSTIFF	SI2		
HASTIFF	SI1/SI2		
HHT	I3	Estimate-correction error is judged by acceleration error	The lowest calculation accuracy and the fastest simulation speed
Newmark	I3

### Classification results and comparison

The validity of the proposed means is proved by five TL tasks of A → B, A → C, A → D, A → E, and B → E. In the first four TL tasks, the training dataset consists of 6000 labeled simulated samples and 3000 unlabeled real samples, and the leftover 600 real samples are used for testing. In TL task B→E, The training dataset consisted of 3000 labeled B dataset samples and 3000 unlabeled E dataset samples, and the remaining 600 E dataset samples were used for testing. CNN, DeepCoral^32^, DDC^33^, DANN^34^ are selected for comparative experiments. In order to compare the accuracy of various means more reasonably, all the above means use the same CNN structure and parameters as the means proposed in this paper.

**Figure 10:**
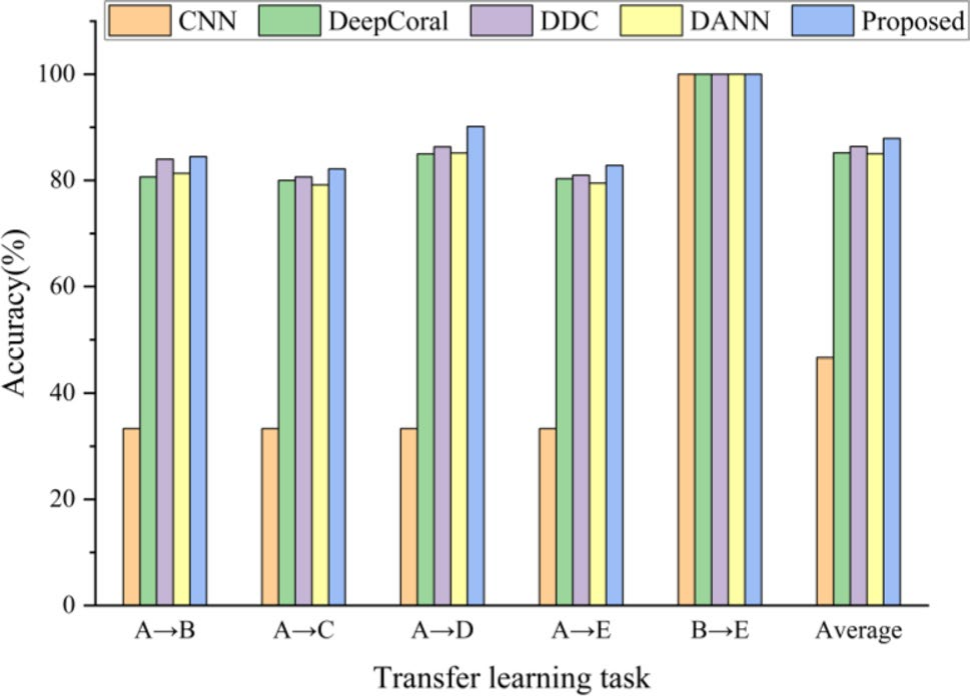
Diagnostic precision of various means on various transfer tasks.

**Figure 11:**
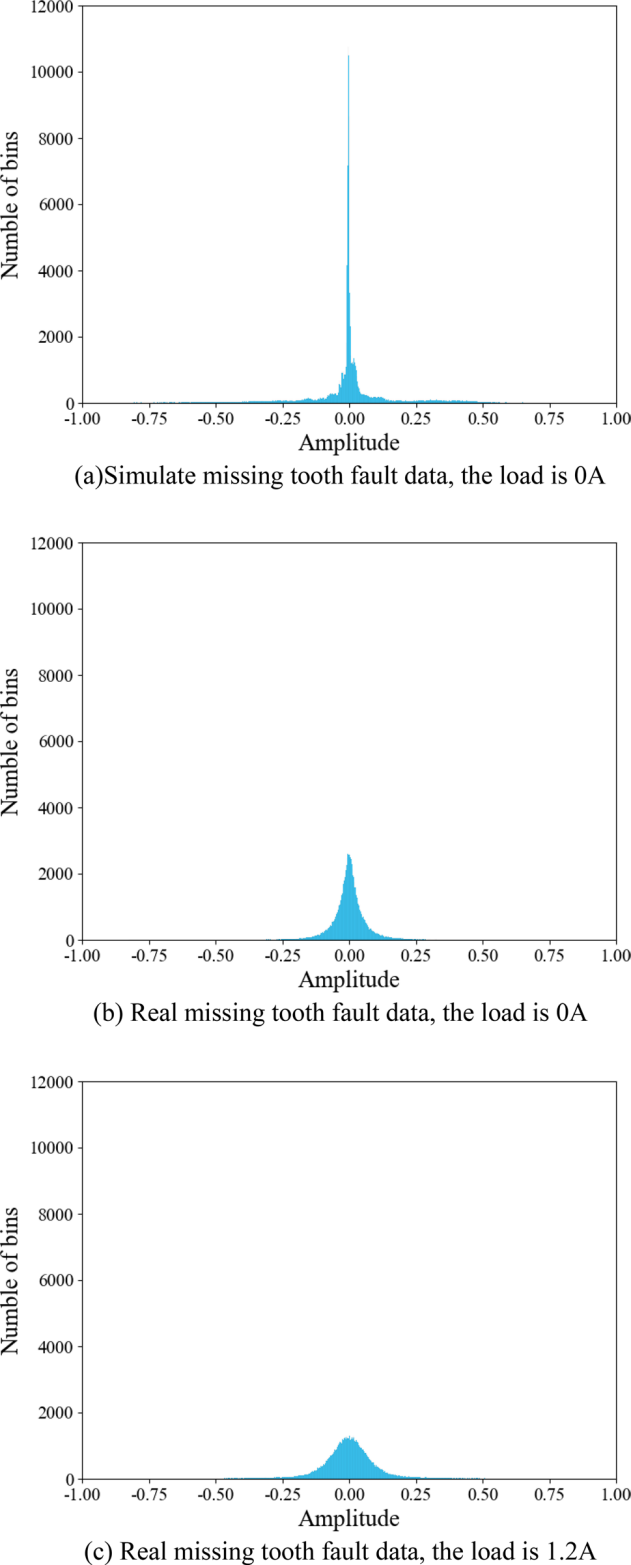
Normal distribution histograms.

Figure 10 displays the result from several diagnostic means. In the four transfer learning tasks, the proposed means obtains the maximum accuracy, demonstrating its effectiveness; Additionally, the accuracy of transfer between real data under various working conditions reaches 100%, which is higher than the accuracy of the transfer from simulation data to real data. The reason is that there is a significant discrepancy between simulation data and real data, while the difference between real data under different working conditions is relatively small. This causes transfer between simulation data and real data to be more challenging and to have poorer accuracy. In order to intuitively show the difference between different data, the normal distribution histograms of the simulated tooth missing tooth fault data with load of 0 A, the real tooth missing tooth fault data with load of 0 A and the real tooth missing tooth fault data with load of 1.2 A are drawn, respectively. It can also be seen from Fig. 11 that the disparities between the simulation data and the real data is far greater than the disparities between the real data under different working conditions.

## Conclusions

To achieve accurate fault diagnosis of PG when marked fault data is insufficient, a fault diagnosis means based on dynamic simulation and DTL is put forward in this paper. The dynamic simulation model of the PG was built by using ADAMS, and the simulation data of various health conditions were obtained. By analyzing the fault simulation data, it is found that the rigid-flexible coupling model can better mirror the fault characteristics of the planetary gear train, and the smaller the simulation step size is, the clearer the periodic impact in TDG and the sidebands in FDG are. By fusing one-dimensional convolutional neural network, attention mechanism, and domain adaptation method, a novel DTL network model is built, and the network model is used to apply diagnostic knowledge from simulation data to real PG’s fault diagnosis. The fault diagnosis experiment was verified by using DDS test bench. By contrasting the diagnosis results of the simulation data with various parameters, it was found that when the integration solver of the simulation data was WSTIFF, the integration format was I3, and the sampling frequency was the same as the real data, the diagnostic accuracy was the highest. The validity of the proposed means is verified by contrasting the diagnostic precision of various means on various transfer tasks.

## Data Availability

The datasets generated and/or analysed during the current study are not publicly available due the data also forms part of an ongoing study, but are available from the corresponding author on reasonable request.
